# Enhancement of Biomass Production in Colony-Forming Green Algae, *Botryosphaerella sudetica*, Under Mixotrophic Cultivation

**DOI:** 10.3389/fgene.2021.669702

**Published:** 2021-06-04

**Authors:** Hyun-Sik Yun, Young-Saeng Kim, Ho-Sung Yoon

**Affiliations:** ^1^Department of Biology, College of Natural Sciences, Kyungpook National University, Daegu, South Korea; ^2^BK21 FOUR KNU Creative BioResearch Group, School of Life Sciences, Kyungpook National University, Daegu, South Korea; ^3^Research Institute of Ulleung-do and Dok-do, Kyungpook National University, Daegu, South Korea

**Keywords:** colony-forming, green algae, mixotrophic cultivation, glucose, settleability, saturated fatty acid, monounsaturated fatty acid

## Abstract

In this study, we characterized the potential of colony-forming green algae, *Botryosphaerella sudetica* KNUA107, isolated from Ulleung Island, South Korea, as a bioresource and analyzed the effects of mixotrophic cultivation on its bioresource production efficiency. Internal transcribed spacer (ITS) (ITS1, 5.8S, and ITS2), ribulose bisphosphate carboxylase large subunit (*rbc*L), and elongation factor Tu (*tufa*) regions were used for molecular identification and phylogenetic analysis. *B. sudetica* KNUA107 had a strong relationship with the green algae of *Botryococcus* and *Botryosphaerella* genera, which are colony-forming species, and was also associated with members of the *Neochloris* genus. To improve biomass productivity, we tested mixotrophic cultivation conditions using several organic carbon sources. Glucose supplementation stimulated *B. sudetica* KNUA107 growth and reduced the time needed to reach the stationary phase. In addition, the colony size was 1.5–2.0 times larger with glucose than in photoautotrophic cultures, and settleability improved in proportion to colony size. The total lipid content and biomass productivity were also higher in cultures supplemented with glucose. Among the lipid components, saturated fatty acids and monounsaturated fatty acids had the highest proportion. Our study suggests that *B. sudetica* KNUA107, which has enhanced efficiency in biomass production and lipid components under mixotrophic cultivation, has high potential as a bioresource.

## Introduction

Green algae are photosynthetic organisms used as bioresources (Vanthoor-Koopmans et al., 2013; Muhammad et al., 2020). They are promising bioenergy feedstocks and can produce more biomass per unit area than terrestrial plants (Cooney et al., 2009; Vanthoor-Koopmans et al., 2013; Bhushan et al., 2020), wherever water, light, and inorganic materials (nitrogen, phosphorous, and trace elements) are available (Smith and Wiedeman, 1964; Cooney et al., 2009; Lu et al., 2020). Usually, lipids compose 10–20% of the biomass produced by green algae (Sajjadi et al., 2018). Despite their high potential as bioenergy feedstocks, there are limitations to the commercial use of green algae (Sajjadi et al., 2018). Specifically, several processes are needed to harvest biomass from culture (centrifugation or filtering) and extract lipids from the harvested biomass (Shelef et al., 1984; Cooney et al., 2009; Li et al., 2020). These processes are expensive and energy-intensive and lead to material losses that decrease productivity (Lee et al., 2010; Halim et al., 2012; Sui et al., 2020). Consequently, the price of the bioenergy produced from green algae is high (Delrue et al., 2012). Therefore, to efficiently produce bioenergy with green algae, it is necessary to reduce the associated costs by improving the process efficiency (Delrue et al., 2012).

Green algae are distributed over a wide range of environments to which they have adapted by evolving distinctive growth habits (Rindi, 2007; Singh and Singh, 2015). For example, some species are multicellular (Marchant, 1974; Coesel and Krienitz, 2007) (e.g., *Pediastrum* genus) and others form colonies that connect single cells (e.g., *Botryococcus* and *Botryosphaerella* genera) (Marchant, 1974; Přibyl and Cepák, 2007). Multicellular species have high settleability and have been used to produce bioresources for bioenergy development (Park et al., 2015). High settleability is important because it allows growing continuous cultures in settling tanks, which reduce harvesting efficiency otherwise (Park et al., 2011, 2015). Harvesting by centrifugation or filtering leads to higher efficiency, but these methods are not suitable for continuous culture (Shelef et al., 1984; Najjar and Abu-Shamleh, 2020). Continuous culture and sediment harvesting can reduce the time and cost associated with bioresource production (Park et al., 2011, 2015; Nazari et al., 2020).

Although green algae are photosynthetic organisms, they can metabolize organic and inorganic carbons (Roostaei et al., 2018). Several organic carbon sources promote algal growth and enhance their lipid content (Roostaei et al., 2018). Hence, mixotrophic culture conditions have been used to increase biomass productivity and lipid accumulation in green algae (Roostaei et al., 2018). Photoautotrophic conditions, which depend only on light energy, are only used in low-density cultures due to the adverse effects of shading as the cell density increases (Goldman, 1979). However, mixotrophic conditions allow high-density cultures, regardless of the shading (Roostaei et al., 2018). In addition, green algae cultured in mixotrophic conditions tend to grow faster and produce more biomass than those grown in exclusively photoautotrophic conditions (Roostaei et al., 2018). Mixotrophic cultures have successfully enhanced biomass productivity and the content of components, including lipids, in various green algae species (Orosa et al., 2001; Ip et al., 2004; Tanoi et al., 2011; Wang et al., 2020). Therefore, mixotrophic culture is a promising approach to reduce costs and increase bioresource productivity using green algae.

In this study, we evaluated the suitability of the colony-forming green alga, *Botryosphaerella sudetica* KNUA107, isolated from Ulleung Island, as bioresources for bioenergy production. To enhance bioresource production, we used mixotrophic cultures supplemented with different organic carbon sources and screened their effect on algal growth. Settleability and lipid contents under various mixotrophic culture conditions were measured and analyzed. Based on these analyses, we identified an organic carbon source that enhanced bioresource productivity and confirmed the value of *B. sudetica* KNUA107 as a bioresource for bioenergy production.

## Results and Discussion

### Identification of the Isolated Algae

**FIGURE 1:**
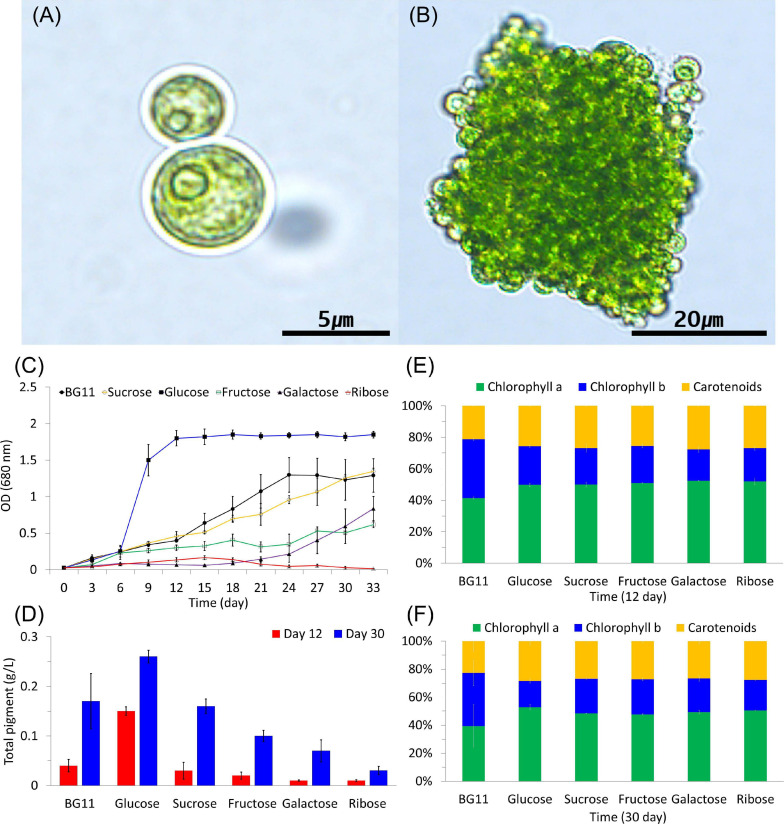
Light microscopic images of *Botryosphaerella sudetica* KNUA107. Under photoautotrophic conditions, *B. sudetica* KNUA107 was observed as single cells **(A)** and colony-forming cells **(B)**. Pigment and chlorophyll contents were measured at 12 and 30 days after growth **(C–F)**.

A green algal strain that formed colonies in liquid medium was among those isolated at Dodong, Ulleung Island (Figure 1). Its cells were approximately spherical and had an average size of 6.26 ± 1.34 μm. The *B. sudetica* KNUA107 strain was assigned the GenBank accession number MW683220 in the NCBI database. *B. sudetica* KNUA107 grew the vegetative cell in the form of single cells and multicellular colonies in the liquid medium, and the colonies were irregular and appeared to be tightly bound between cells (Figure 1). The blast results (NCBI database) of the *B. sudetica* KNUA107 marker gene sequences are summarized in Supplementary Table 1. The sequence analyses suggested that *B. sudetica* KNUA107 was a species similar to *Botryococcus* sp., *Botryosphaerella sudetica*, and *Neochloris aquatica* (Supplementary Table 2). It had the highest sequence similarity with *B. sudetica* (95%), followed by *Botryococcus* sp. (92%) and *N. aquatica* (91%). Previous data indicated that some members of these three green algae genera have colony-forming abilities and morphological characteristics similar to those of *B. sudetica* KNUA107 (Komárek, 1989; Senousy et al., 2004; Přibyl and Cepák, 2007; Tanoi et al., 2011). *B. sudetica* was recently established as a new taxon after being previously named *Botryococcus sudeticus* (Senousy et al., 2004; Přibyl and Cepák, 2007). *Botryosphaerella* cell morphology differs from that of *Botryococcus* and is more similar to that of *Neochloris* (Senousy et al., 2004; Přibyl and Cepák, 2007). However, colonies formed by *Botryosphaerella* and *Neochloris* differ in morphology and characteristics (Komárek, 1989; Přibyl and Cepák, 2007). Hence, the members of the three genera are distinguishable by colony morphology (Komárek, 1989; Senousy et al., 2004; Přibyl and Cepák, 2007; Tanoi et al., 2011). The cell morphology of *B. sudetica* KNUA107 was similar to that of green algae in *Botryosphaerella* and *Neochloris* genera (Figure 1A). Additionally, its colony morphology was similar to that of *Botryosphaerella* (Figure 1B).

Chlorophyll and total pigment contents were measured based on the growth kinetics of cultures under photoautotrophic and mixotrophic conditions (Figures 1C–F). Chlorophyll is also included in extractable pigments, and carotenoid pigments are also part of the measured all pigments. In previous studies, various environmental conditions were measured based on growth kinetics, all pigment, and chlorophyll contents (Schuurmans et al., 2015; Tubuxin et al., 2015; Sivakaminathan et al., 2018). The selected strains grew better than the control strains under various environmental conditions. As a result, the selected strains showed an increase of all pigment and chlorophyll than the control strains (Kim et al., 2018, 2020). After 6 days of growth, chlorophyll and pigment contents were considerably increased in the cultures supplemented with glucose compared with all other conditions. *B. sudetica* KNUA107 also recovered growth quickly when supplemented with glucose (6 days; Figure 1C) and reached higher survival rates than when grown under other mixotrophic conditions. After 12 and 30 days of glucose supplementation, chlorophyll and total pigment contents increased approximately twofold compared with those under other mixotrophic treatments (Figures 1C–F).

**FIGURE 2:**
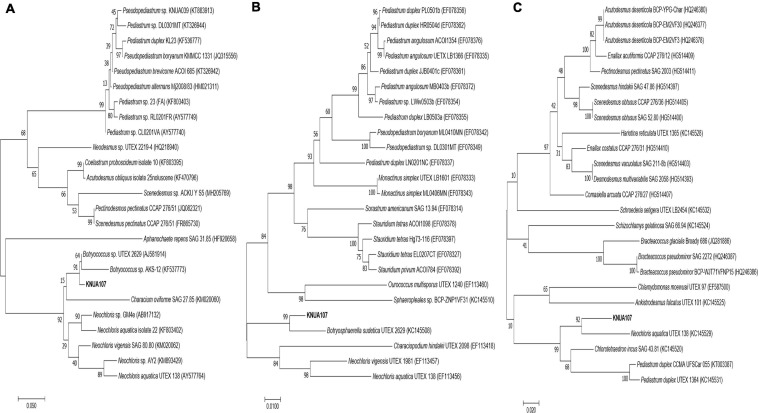
Molecular phylogenetic analysis of *B. sudetica* KNUA107 according to the Maximum Likelihood (ML) tree. Numbers at the nodes indicate bootstrap probabilities of ML analyses (1,000 replicates). Three marker genes were used for the analysis: the ITS (internal transcribed spacer) region **(A)**, the *rbc*L (large unit ribulose bisphosphate carboxylase) region **(B)**, and the *tuf*A (elongation factor Tu gene) region **(C)**.

The relationship between *B. sudetica* KNUA107 and the three similar green algae species (*Botryococcus* sp., *B. sudetica*, and *N. aquatica*) was further determined by phylogenetic analysis. ML trees were constructed based on ITS, *rbcl*, and *tufa* sequences of *B. sudetica* KNUA107 and several green algal species (Figure 2). Based on each gene sequence, *B. sudetica* KNUA107 belonged to distinct groups that included *Botryococcus*, *Botryosphaerella*, or *Neochloris*. The analysis revealed that *B. sudetica* KNUA107 had the closest phylogenetic relationship with the three strains to which it had high sequence similarity. *B. sudetica* had the highest bootstrap probabilities with *B. sudetica* KNUA107, reaching 99% (*Botryococcus* sp., 91%; *N. aquatica*, 92%). Hence, *B. sudetica* KNUA107 had the closest phylogenetic relationship with *B. sudetica*. Additionally, *B. sudetica* KNUA107 has a colony-forming ability similar to previously studied *B. sudetica* algae (Figures 1A,B). These results confirm *B. sudetica* KNUA107 is a *B. sudetica* strain.

### Algal Growth and Biomass Production

**FIGURE 3:**
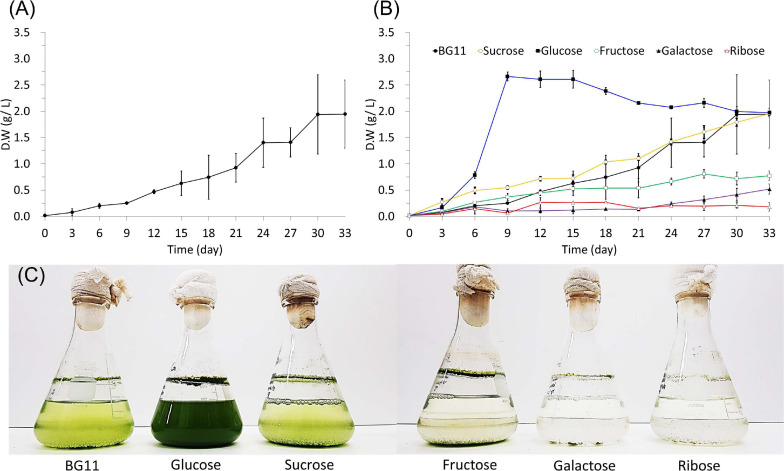
Growth of *B. sudetica* KNUA107 under photoautotrophic (BG11) **(A)** and mixotrophic (sucrose, glucose, fructose, galactose, and ribose) **(B)** conditions measured by dry weight. Visible changes in KNUA107 cultures with each culture condition at day 30 **(C)**.

The dry weight was measured to determine the growth and biomass production of cultured *B. sudetica* KNUA107 (Figure 3). Algae cultured with photoautotrophic conditions had a long growth period and took more than 30 days to reach the stationary phase; the maximum biomass productivity obtained was 1.94 ± 0.75 g/L (Figure 3A). This result suggested that *B. sudetica* KNUA107 required a longer cultivation time than other green algae species to achieve biomass production (Singh and Singh, 2015; Metsoviti et al., 2020). However, after 30 days of culture, the biomass produced was higher than for other green algae species after the same growth period (Singh and Singh, 2015). Next, we investigated if mixotrophic cultivation with five organic carbon sources (up to 40 mM of sucrose, glucose, fructose, galactose, and ribose) increased biomass productivity in *B. sudetica* KNUA107 by analyzing the growth patterns and biomass productivity under each condition (Figure 3B). In previous studies, glucose has been used of carbon sources in the form of polysaccharides in biomass yield productivity than acetate. In the comparison of glucose, ethanol, and acetate as a carbon source for poly(3-hydroxybutyrate; PHB) production and biomass yield productivity, glucose and ethanol is coupled with the generation of energy and inducing power, but extra acetate is oxidized to meet the demand of energy and reducing power in the pathway from acetate to PHB and biomass yield productivity. Therefore, organic carbon sources such as sucrose, fructose, galactose, and ribose were used in our overall studies (Johnson and Alric, 2013; Sun et al., 2020). Only glucose supplementation increased the growth and biomass productivity of cultured algae. The maximum biomass production, 2.66 ± 0.04 g/L, was obtained after 9 days of culture. Three of the tested organic carbon sources, fructose, galactose, and ribose, inhibited the growth and biomass productivity of cultured algae, leading to maximum values of 0.81 ± 0.08 g/L, 0.52 ± 0.10 g/L, and 0.27 ± 0.09 g/L, respectively, all lower than that of the photoautotrophic condition (1.94 ± 0.75 g/L). Supplementation with sucrose led to growth and maximum biomass production (1.96 ± 0.08 g/L) similar to those of the photoautotrophic condition. The effects of mixotrophic cultivation on biomass productivity were also evident by observing the changes in algal culture growth (Figure 3C). The cell density of algal cultures is proportional to the measured dry weight (Sutton, 2011; Tejido-Nuñez et al., 2020). Therefore, we attempted to quantify algal cell density with OD measurements, but there were limitations to this approach (Kargul et al., 2003; Beal et al., 2020). The algal culture supplemented with glucose exceeded the maximum measurement range of optical density (2.000). However, algal cultures grown under photoautotrophic conditions (1.698 ± 0.039) and other mixotrophic conditions (sucrose, 1.625 ± 0.016; fructose, 0.722 ± 0.013; galactose, 0.389 ± 0.036; and ribose, 0.313 ± 0.015) had OD_680_ values proportional to the dry weight. Although there was a limit in measuring the OD value of algal cultures, we expect that *B. sudetica* KNUA107 can be cultured at a high density in glucose mixotrophic conditions (Goldman, 1979; Verma et al., 2020).

The biomass produced by *B. sudetica* KNUA107 cultures was higher than for other green algae species but required longer culture cycles (Singh and Singh, 2015). Because short culture cycles are one of the advantages of biomass production using green algae (Forján et al., 2015), the long culture required by *B. sudetica* KNUA107 was a limitation that needed to be addressed. Thus, we tested mixotrophic cultivation conditions to promote green algae growth and improve biomass productivity (Cheirsilp and Torpee, 2012; Patel et al., 2020). Glucose supplementation enhanced the growth and biomass productivity and decreased the culture cycle from 30 to 9–12 days; the biomass productivity increased by 0.72 g/L. Previously, *B. sudetica* growth was enhanced under mixotrophic conditions due to the addition of organic carbon source (Zhang et al., 2021), as confirmed by comparison with previous studies on other microalgae (Meng et al., 2020; Smith et al., 2021). In contrast, only glucose promoted *B. sudetica* KNUA107 growth, and some organic carbon sources inhibited it (Heredia-Arroyo et al., 2011). A previous study reported the negative effect of osmotic stress on mixotrophic cultivation of other microalgae (Ryu et al., 2020). Osmotic stress can inhibit microalgae growth and alter gene expression (Zhou et al., 2020; Chuang and Liao, 2021). Therefore the organic carbon sources directly available to *B. sudetica* KNUA107 might have been limited due to osmotic stress (Yeesang and Cheirsilp, 2014). These results suggest that mixotrophic cultivation can shorten the culture cycle of green algae and improve biomass productivity. However, not all organic carbon sources promote green algae growth and may instead inhibit it through mechanisms, including the effects of osmotic stress.

### Colony-Forming Ability and Settleability

**FIGURE 4:**
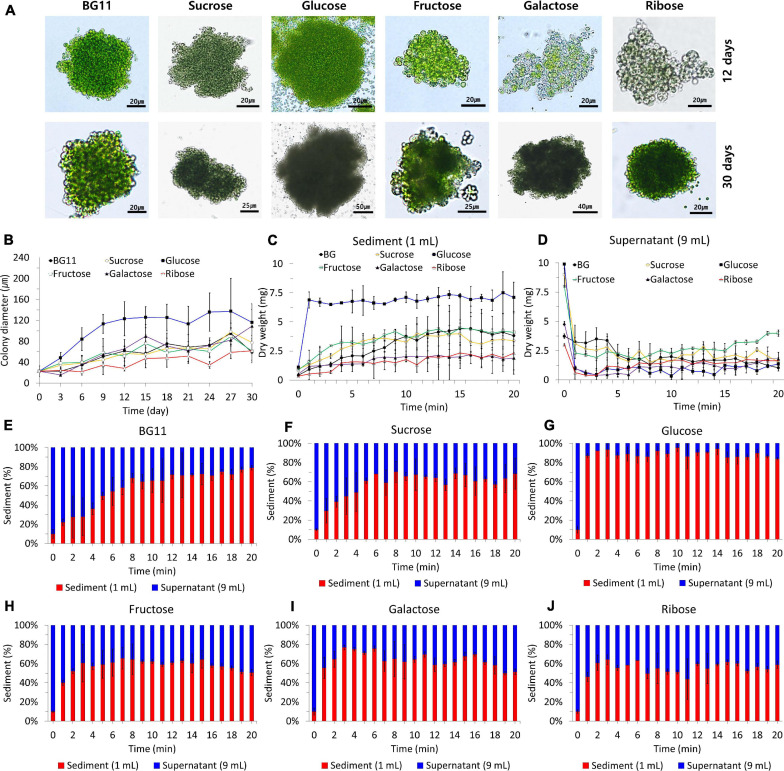
*B. sudetica* KNUA107 colonies **(A)** and their size recorded in each culture condition during the culture process **(B)**. Settleability of *B. sudetica* KNUA107 cultured under each condition evaluated by the dry weight of the sediment **(C)** and the supernatant **(D)**. Sedimentation rate for photoautotrophic (BG11) **(E)** and mixotrophic conditions, including sucrose **(F)**, glucose **(G)**, fructose **(H)**, galactose **(I)**, and ribose **(J)**. The results are expressed as the ratio of the biomass, which is defined as 100%.

We investigated the colony-forming ability and settleability of *B. sudetica* KNUA107 cultured in photoautotrophic and mixotrophic conditions (Figure 4). To determine changes in colony-forming ability, we measured the diameters of algal colonies formed under each condition (Figures 4A,B). The carbon sources added to cultivation media influenced the average colony sizes. The largest colonies occurred in the presence of glucose (137.25 ± 36.57 μm), and the smallest colonies occurred with ribose supplementation (61.84 ± 22.93 μm). The colony size obtained with other mixotrophic conditions and with the photoautotrophic condition (BG11), averaged 80–100 μm (BG11, 95.12 ± 24.62 μm; sucrose, 98.23 ± 16.67 μm; fructose, 89.42 ± 16.30 μm; and galactose, 82.42 ± 22.73 μm). The average size of colonies stopped increasing at 9–12 days of cultivation with glucose and at 27–30 days for other conditions, similar to that of supplemented condition (Figure 3). Furthermore, the pattern of colony size increase for each culture condition was similar to the normal *B. sudetica* KNUA107 growth.

The settleability of colonies formed under each condition was estimated using the sediment dry weight (Figures 4C–J). The sediment dry weight increased over time for all culture conditions (Figures 4C,D). In the cultures supplemented with glucose, the dry weight stopped increasing 1 min after the start of the measurement period. Under the other growth conditions, the dry weight stopped increasing between 3 and 8 min (BG11, 8 min; sucrose, 6 min; fructose, 3 min; galactose, 3 min; and ribose, 3 min). All mixotrophic algal cultures had faster sedimentation rates than the photoautotrophic cultures. With glucose as an organic carbon source, *B. sudetica* KNUA107 sedimentation was completed approximately 7 min faster than in the photoautotrophic condition. Cultures supplemented with glucose had the highest proportion of the settled biomass, reaching 84.02–95.47% of the total biomass. However, the proportion of the settled biomass was less than 80% in other culture conditions, reaching 64.37–78.74% with BG11, 56.86–70.47% with sucrose, 43.75–64.20% with fructose, 51.35–76.84% with galactose, and 50.69–65.47% with ribose supplementation (Figures 4E–J).

We also observed variations in algal cell size during the measurements of colony size (Figure 4A). In the photoautotrophic condition, *B. sudetica* KNUA107 cells averaged 6.26 ± 1.34 μm, but size decreased in mixotrophic conditions, reaching 5.32 ± 1.56 μm with sucrose, 3.44 ± 0.48 μm with glucose, 4.52 ± 0.79 μm with fructose, 3.94 ± 0.15 μm with galactose, and 3.58 ± 0.47 μm with ribose. Although glucose supplementation led to the smallest cells, it also produced the largest colonies, averaging 137.25 ± 36.57 μm.

Colony size is one of the main factors that affect settleability (Park et al., 2011, 2015; Su et al., 2012; Fan et al., 2020). Settleability changed with mixotrophic conditions, increasing with glucose and decreasing with other added carbon sources. In addition, the growth patterns under different conditions mimicked the changes in colony size. These results showed that the growth and colony-forming ability of *B. sudetica* KNUA107 were similarly enhanced by glucose supplementation. The sedimentation rate and proportion of the settled biomass were used as a standard for evaluating settleability. The fastest sedimentation rate and the highest proportion of the settled biomass were measured in the mixotrophic condition with glucose. Other conditions tended to be similar, with no noticeable difference. Based on the sedimentation rate and proportion of settled biomass, we confirmed that changes in the colony size induced by the mixotrophic conditions were related to changes in settleability (Park et al., 2011, 2015; Su et al., 2012; Fan et al., 2020). Therefore, these results suggest that the mixotrophic condition supplemented with glucose enhances the colony-forming ability and settleability of *B. sudetica* KNUA107. These results support the use of mixotrophic conditions to increase the bioresource harvesting efficiency in this strain.

### Lipid Productivity and Contents From Biomass Produced

**FIGURE 5:**
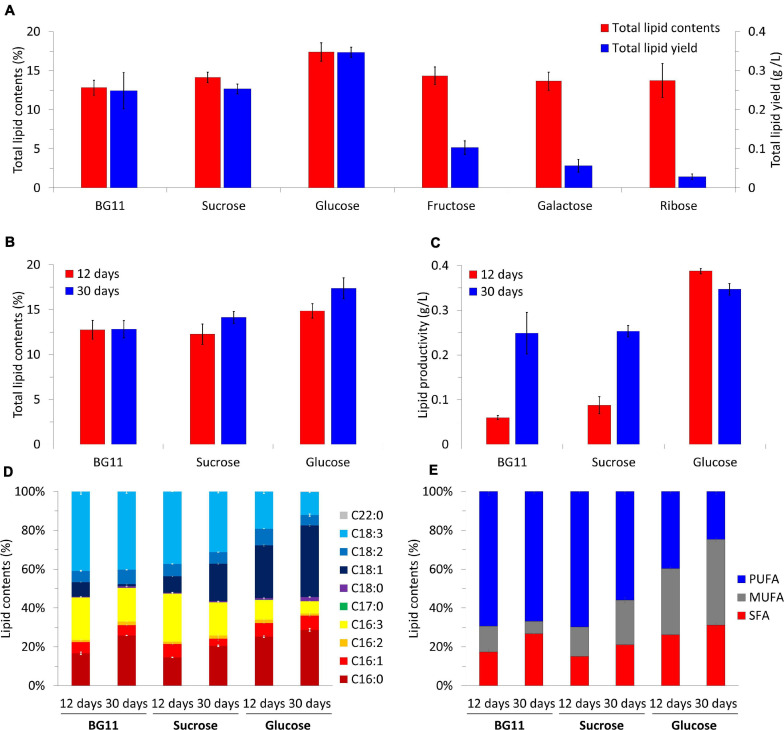
Total lipid contents and total lipid yields of *B. sudetica* KNUA107 under photoautotrophic (BG11) and mixotrophic (sucrose, glucose, fructose, galactose, and ribose) conditions **(A)**. Comparison of harvested biomass at days 12 and 30 under three conditions with high lipid productivity, including total lipid contents **(B)**, lipid productivity **(C)**, fatty acid composition **(D)**, and fatty acid saturation ratio **(E)**. The results are expressed as the ratio of lipid and fatty acid contents, which are defined as 100%. Marked characteristics are annotated in Supplementary Table 2.

We analyzed the lipid productivity and quality of the biomass under mixotrophic cultivation (Figure 5). The biomass harvested at day 30 had 12–18% total lipid contents (Figure 5A). The value was the lowest under photoautotrophic conditions (12.83 ± 0.96%). Although the biomass under mixotrophic conditions had higher total lipid contents than that of the photoautotrophic condition (sucrose, 14.13 ± 0.67%; glucose, 17.39 ± 1.17%; fructose, 14.34 ± 1.13%; galactose, 13.66 ± 1.15%; and ribose, 13.74 ± 2.16%), only glucose led to a noticeable increase in that value. The lipid yields were calculated based on the results of the total lipid contents (Figure 5A). The lipid yield was lower in mixotrophic conditions with fructose (0.10 ± 0.02 g/L), galactose (0.06 ± 0.02 g/L), and ribose (0.03 ± 0.01 g/L) than in the photoautotrophic and sucrose condition, both with 0.25 ± 0.05 g/L). Only glucose supplementation increased lipid yield value, averaging 0.35 ± 0.01 g/L.

Because *B. sudetica* KNUA107 reached the stationary phase on day 12 in the glucose mixotrophic condition (Figure 3B), we analyzed the lipid productivity of the biomass harvested on that day (Figures 5B,C). There was no significant difference between total lipid contents on days 12 (12.76 ± 1.04%) and 30 (12.83 ± 0.96%) in the photoautotrophic condition. In contrast, total lipid contents were higher on day 30 (sucrose, 12.27 ± 1.13%; and glucose, 14.86 ± 0.81%) than on day 12 (sucrose, 14.13 ± 0.67%; and glucose, 17.39 ± 1.17%) under mixotrophic conditions. Lipid productivity was higher at day 30 than at day 12 with the photoautotrophic condition (day 12, 0.06 ± 0.01 g/L; and day 30, 0.25 ± 0.05 g/L) and with sucrose (day 12, 0.09 ± 0.02 g/L; and day 30; 0.25 ± 0.01 g/L). Conversely, in the mixotrophic condition supplemented with glucose, the lipid productivity was higher at day 12 (0.39 ± 0.01 g/L) than at day 30 (0.35 ± 0.01 g/L). The total lipid content and lipid productivity were the highest in the mixotrophic condition supplemented with glucose regardless of the culture period. Moreover, the condition with high biomass productivity also had high lipid productivity.

Next, we analyzed the lipid content composition of biomass with enhanced lipid productivity (Figures 5D,E and Supplementary Table 2). The fatty acids extracted from the biomass under the photoautotrophic condition mainly consisted of C16:0 (day 12, 15.64 ± 0.68%; and day 30, 24.54 ± 0.06%), C16:3 (day 12, 20.57 ± 0.32%; day 30;, 16.42 ± 0.04%), and C18:3 (day 12, 38.38 ± 1.34%; and day 30, 38.23 ± 0.89%). Additionally, C16:0 increased, and C16:3 decreased as the culture progressed. The fatty acid content of the biomass obtained under the mixotrophic condition supplemented with sucrose was mainly composed of C16:0 (day 12, 14.11 ± 0.08%; and day 30, 18.98 ± 0.38%), C16:3 (day 12, 23.90 ± 0.68%; and day 30, 15.82 ± 0.17%), C18:1 (day 12, 8.12 ± 0.11%; and day 30, 17.91 ± 0.34%), and C18:3 (day 12, 36.01 ± 0.01%; and day 30, 28.92 ± 0.68%), and the proportions were similar to those of the photoautotrophic condition. However, as the culture progressed, C18:1 increased, and C18:3 decreased under sucrose. Glucose led to fatty acids with distinct characteristics; they mainly consisted of C16:0 (day 12, 24.71 ± 0.40%; and day 30, 27.66 ± 0.74%), C18:1 (day 12, 26.55 ± 0.61%; and day 30, 35.42 ± 0.51%), and C18:3 (day 12, 18.72 ± 0.97%; and day 30, 11.47 ± 0.33%) and contained the highest proportion of C16:0 and C18:1 among all samples analyzed. In addition, C18:3, which ranged 25–40% under other conditions, was <20% in the glucose condition. The fatty acids analyzed were classified into saturated fatty acids (SFA; C16:0, C17:0, C18:0, C20:0, and C22:0), monounsaturated fatty acids (MUFA; C16:1 and C18:1), and polyunsaturated fatty acids (PUFA; C16:2, C16:3. C18:2, and C18:3), and these classes were compared (Figure 5C and Supplementary Table 2). In the photoautotrophic condition, there was little change in PUFA between days 12 and 30 (day 12, 69.35 ± 2.11%; and day 30, 66.85 ± 1.13%), whereas MUFA decreased (day 12, 13.24 ± 0.13%; and day 30; 6.43 ± 0.20%) and SFA increased (day 12, 17.42 ± 1.05%; and day 30, 26.72 ± 0.07%). In contrast, PUFA decreased with the mixotrophic conditions supplemented with sucrose (day 12, 69.73 ± 0.81%; and, day 30, 55.91 ± 1.32%) and glucose (day 12, 39.67 ± 1.25%; and day 30, 24.63 ± 1.37%); MUFA under sucrose (day 12, 15.24 ± 0.41%; and day 30, 23.01 ± 1.34%) and glucose (day 12, 34.15 ± 0.79%; and day 30, 44.18 ± 0.98%) and SFA under sucrose (day 12, 15.03 ± 0.11%; and day 30, 21.08 ± 0.39%) and glucose (day 12, 26.18 ± 0.48%; day 30, 31.19 ± 0.98%) tended to increase as the culture progressed. However, the PUFA content under sucrose supplementation was higher than 50%, which was similar to the content under photoautotrophic conditions and different from that obtained under the glucose condition. The lowest PUFA and highest MUFA and SFA contents were observed in the mixotrophic condition supplemented with glucose. Glucose was also the only carbon source that led to MUFA and SFA contents higher than the PUFA contents.

Lipids are useful substances that can be obtained by using green algae as a bioresource (Priyadarshani and Rath, 2012; Vanthoor-Koopmans et al., 2013). Previously, biotic and abiotic stresses were applied to green algae to enhance the lipid content of a limited available biomass (Rashid et al., 2020; Ananthi et al., 2021). For example, control of the nitrogen supply and type of sources (e.g., nitrate) increased the ratio of lipids to the weight unit (Dixit et al., 2020; Encinas-Arzate et al., 2020; Zhang et al., 2020). Nevertheless, increased lipid content was not accompanied by a positive effect on biomass productivity (Araujo et al., 2020; Toumi and Politaeva, 2021). By applying a mixotrophic condition that provided an organic carbon source, it was possible to further increase lipid productivity under biotic and abiotic stress (Patel et al., 2020; Laraib et al., 2021). Furthermore, microalgae biomass and lipid production could be enhanced under mixotrophic cultivation (Cheirsilp and Torpee, 2012). Here, we cultured *B. sudetica* KNUA107 under mixotrophic conditions supplemented with glucose and enhanced its lipid contents and lipid productivity. In addition, we confirmed that the fatty acids increased under mixotrophic conditions were mostly of the saturated type. Our research suggests that applying mixotrophic conditions to *B. sudetica* KNUA107 culture can improve lipid productivity. In addition, by providing glucose as an organic carbon source, it is possible to produce high-quality fatty acids with enhanced MUFA and SFA contents.

## Conclusion

In this study, we isolated a colony-forming green algae strain at Ulleung Island (*B. sudetica KNUA107*) and confirmed that it is related to *B. sudetica*. We evaluated biomass and lipid productivity to demonstrate its potential as a bioresource for bioenergy production. Furthermore, we applied mixotrophic cultivation, which promotes bioenergy production in microalgae, to *B. sudetica* KNUA107. Several mixotrophic conditions were applied to the culture to investigate their effect on biomass productivity. Glucose was the only added carbon source that increased biomass in high-density cultures, increasing growth rate and settleability. Moreover, the glucose mixotrophic condition increased *B. sudetica* KNUA107 content in lipids and their saturated components. Therefore, *B. sudetica* KNUA107 is a suitable strain to produce bioresources under mixotrophic conditions. Our research elucidates the impact of organic carbon sources on biomass productivity and suggests the potential of colony-forming green algae as an industrially useful bioresource.

## Materials and Methods

### Sampling and Isolation of Microalgae

For the isolation and screening of green algae, a freshwater sample was collected from Dodong in Ulleung Island, South Korea (37°29′51.5″N, 130°53′16.1″E) in April 2017. The sample was transferred to the laboratory and inoculated in 150 mL of BG11 medium (pH 7.4) in a 250 mL flask (Rippka et al., 1979). The inoculated sample was incubated at 25°C, under a fluorescent lamp (approximately 55 μmol m^–2^ s^–1^), and with a 16:8 h light:dark cycle. The culture was rotated at 160 rpm in an orbital shaker (VS-202D, Vision Scientific, Bucheon, South Korea). After a 2-week incubation, the algal sample was streaked onto a BG11 (pH 7.4) agar plate. Subsequently, single colonies grown were aseptically transferred to fresh BG11 agar plates. The transferring process was repeated until a colony consisting of one species was aseptically obtained (Stanier et al., 1971).

### Molecular Identification

For the molecular identification of aseptically isolated *B. sudetica* KNUA107 strains, cells that were incubated for 2 weeks in 150 mL of BG11 were collected by centrifugation at 4000 rpm for 10 min and washed twice with sterile distilled water. The collected algal cells were resuspended in 400 μL DNA extraction buffer (100 mM Tris HCl, 1 M KCl, and 10 mM EDTA) in 2 mL microfuge tubes. The suspended samples were vortexed for 5 min after adding 8 μL RNAse A (50 mg/mL, Elpis-Biotech, Daejeon, South Korea) and sterile glass beads and heated at 65°C for 30 min. Cellular debris was removed by centrifugation at 13,000 rpm for 10 min, and 400 μL of the supernatant was transferred into a new tube. DNA was purified with a DNA purification kit (Wizard DNA Clean-Up System, Promega, Madison, WI, United States) according to the manufacturer’s protocol. The internal transcribed spacer (ITS), ribulose bisphosphate carboxylase large subunit (*rbc*L), and elongation factor Tu (*tufa*) were used as marker genes (White et al., 1990; Famà et al., 2002; Saunders, 2010; McManus and Lewis, 2011). The ITS region was amplified with one primer set (ITS5 F, 5′-GGAAGTAAAAGTCGTAACAAGG-3′, and ITS5 R, 5′-TCCTCCGCTTATTGATATGC-3′) (White et al., 1990), the *rbc*L region was amplified with two primer sets (rbcL-M379-F, 5′-GGTTTCAAAGCTYTWCGTGC-3′, and rbcLFP-R, 5′-GTAAATACCACGGCTACGRTCTT-3′; and GrbcL-F, 5′-GCTG GWGTAAAAGATTAYCG-3′, and GrbcL-R, 5′-TCACGCC AACGCATRAASGG-3′) (Saunders, 2010; McManus and Lewis, 2011), and the *tuf*A region was amplified with one primer set (tufA-F, 5′-GGNGCNGCNCAAATGGAYGG-3′, and tufA-R, 5′-CCTTCNCGAATMGCRAAWCGC-3′) (Famà et al., 2002). The amplified DNA fragments were purified with a PCR Purification kit (Spin type-200, Elpis-Biotech, Daejeon, South Korea) according to the manufacturer’s protocol. For DNA sequencing, DNA fragments were purified with the pGEM^®^ -T Easy Vector kit (Promega, Madison, WI, United States). DNA sequencing was conducted at the Genotech facility (Genotech, Daejeon, South Korea). The sequence information of previously reported related algal strains (with similar sequences) was retrieved from NCBI; *B. sudetica* KNUA107 collected from Ulleung islands received the GenBank accession number MW683220. The selected sequences were aligned using the MEGA (version 7.0) software package to analyze the phylogenetic relationship between algal strain (Kumar et al., 2016). Based on the Bayesian information criterion, the best-fit nucleotide substitution model of each marker gene was selected for analysis. A maximum likelihood (ML) phylogenetic tree was built with 1,000 bootstrap replications (Felsenstein, 1985).

### Photoautotrophic and Mixotrophic Cultivation

A 30-day-old *B. sudetica* KNUA107 culture was prepared for photoautotrophic and mixotrophic cultivation. The algal cells were cultured with shaking at 160 rpm in an illuminated incubation room at 25°C. The prepared algal cells were diluted to approximately 156.67 ± 20.81 mg/L. BG11 medium was used for photoautotrophic cultivation (Rippka et al., 1979), and organic carbon sources up to 40 mM of sucrose, glucose, fructose, galactose, and ribose were added to BG11 for mixotrophic cultivation. After each medium was sterilized, 150 mL was transferred to a 250 mL flask and inoculated with 15 mL of the prepared algal cells. Flasks were cultured at 25°C in an orbital shaker (160 rpm) in an incubation room illuminated with approximately 55 μmol m^–2^ s^–1^ light intensity and a 16:8 h light:dark cycle.

### Growth and Pigment Content Analysis

The growth of *B. sudetica* KNUA107 was estimated using the dry weight (Yoo et al., 2010). Samples (3 mL) were collected from culture flasks at 3 day intervals, filtered with a pre-weighed glass fiber filter, and washed with distilled water (Yoo et al., 2010). The filtered algal cell pellet was dried in a dry oven at 105°C for 1 day and then weighed (Yoo et al., 2010). Chlorophyll content assays were conducted by harvesting algal cells from 100 mL cultures at OD_680_. The cultivated cells were harvested by centrifugation, and the pellets were resuspended in 90% methanol. Chlorophyll was extracted as previously described (Kim et al., 2018, 2020) with some modifications. All experiments were performed at least three times independently. For total pigment content measurements, samples were sonicated, and the pigments were extracted with Methanol (Şükran et al., 1998). The OD_666_, OD_653_, and OD_470_ values of the extracts were measured with a spectrophotometer (Lichtenthaler and Wellburn, 1985). Pigment content was calculated from the measured OD values according to the formulas of Lichtentaler and Wellburn (Lichtenthaler and Wellburn, 1985). Pigment and chlorophyll contents were measured at 12 and 30 days after approximately 1 month of growth.

### Settleability Analysis

Colony size, a phenotype associated with settleability, was measured (Park et al., 2011, 2015) in samples collected in 3-day intervals, starting from day 0. Colony diameter was measured using an optical microscope (Nikon Eclipse E100 Biological Microscope, Tokyo, Japan). To compare the settleability of *B. sudetica* KNUA107 cultured under each tested condition, 30-day-old samples were collected by centrifugation. Samples were sufficiently resuspended, and 10 mL of each was transferred into 15 mL conical tubes. A set of 21 conical tubes was used to measure the settleability for each culture condition. Prior to measurement, one set of tubes was inverted simultaneously, and one tube was measured per min by separating 9 mL of the supernatant from the sample and separately measuring the dry weight of the algal cells contained in the supernatant (9 mL) and sediment (1 mL). The measurements were performed for 20 min per set, with three sets per culture condition.

### Biomass Collection and Lipid Contents Analyses

For lipid content analysis, algal biomass was collected at the middle (12-day-old) and at the end (30-day-old) of the cultivation period by centrifugation at 4,000 rpm for 30 min. The collected cells were then freeze-dried. The sulfo-phospho-vanillin colorimetric method was used to determine the total lipid content (Mishra et al., 2014). Canola oil was used to generate the equations and standard curve. Finely ground freeze-dried samples (10 mg) were placed in fresh tubes and resuspended in 1 mL of distilled water. Different volumes (10, 20, 30, 40, and 50 μL) of the resuspended samples were added to glass tubes, which were then filled up to 100 μL with distilled water. Sulfuric acid (2 mL) was added to each glass tube, and the mixture was heated in a water bath at 100°C for 5 min. After heating, the samples were cooled in ice for 5 min, and 5 mL of the phospho-vanillin reagent was added to each tube. The mixtures were shaken at 200 rpm for 15 min in an incubator at 37°C. The optical density at 530 nm (OD_530_) was measured in mixtures with sufficient reaction, and the total lipid content was calculated based on the measured value (Mishra et al., 2014).

Lipids were extracted and analyzed by gas chromatography/mass spectrometry (GC/MS) to determine their composition and content (Yeo et al., 2011; Furuhashi and Weckwerth, 2013). Freeze-dried samples (30 mg) were pulverized, and the lipids in the sample were extracted using chloroform:methanol (1:1) (Yeo et al., 2011). Chloroform was removed from the extracted mixture using a rotary evaporator. To facilitate lipid transesterification, the extracted mixture was treated with a methanol and potassium hydroxide reagent. Hexane was added to the mixture for the isolation of fatty acid methyl esters (FAME). The reaction mixture was heated in a water bath at 70°C for 3 h. As an external standard for calculating the FAME contents, the hexane layer was analyzed using GLC-90 (SUPELCO, Bellefonte, PA, United States). The internal standards used helium as the carrier, and the fatty acid composition was analyzed using the W11N17MAIN chemical library database instead of the standard material. A 6890N gas chromatograph (Agilent Technologies Inc., Santa Clara, CA) equipped with a 5973N mass selective detector (Agilent Technologies Inc., Santa Clara, CA) and HP-5MS capillary column (30 m × 0.25 mm ID × 0.25 μm film thickness; Agilent Technologies Inc., Santa Clara, CA) was used to analyze the lipids contained in FAME (Furuhashi and Weckwerth, 2013).

## Data Availability Statement

The datasets presented in this study can be found in online repositories. The names of the repository/repositories and accession number(s) can be found in the article/Supplementary Material.

## Author Contributions

H-SYu: investigation and writing—original draft. Y-SK: investigation. H-SYu and Y-SK: conceptualization, data curation, formal analysis, and methodology. H-SYu: supervision. Y-SK and H-SYo: funding acquisition. H-SYu, Y-SK, and H-SYo: writing—review and editing. All authors contributed to the article and approved the submitted version.

## Conflict of Interest

The authors declare that the research was conducted in the absence of any commercial or financial relationships that could be construed as a potential conflict of interest.

## Supplementary Material

Supplementary Table 1 BLAST sequence alignment output results using marker genes (ITS, *rbc*L, *tuf*A) of *B. sudetica* KNUA107.

Supplementary Table 2 Fatty acid productivity and composition of *B. sudetica* KNUA107 under different mixotrophic cultivation conditions.

## Abbreviations

ITS: internal transcribed spacer
SFA: saturated fatty acid
MUFA: monounsaturated fatty acid
PUFA: polyunsaturated fatty acid
