# Supplementary material for: Enhancement of Biomass Production in Colony-Forming Green Algae, Botryosphaerella sudetica, Under Mixotrophic Cultivation
Source: Front Genet. 2021 Jun 4;12:669702. doi: 10.3389/fgene.2021.669702 (PMC8212961; doi:10.3389/fgene.2021.669702)
Supplement: Supplementary Table 1 — BLAST sequence alignment output results using marker genes (ITS, rbcL, tufA) of B. sudetica KNUA107. [file Data_Sheet_1.PDF]

Supplementary Material

Supplementary Tables

SUPPLEMENTARY TABLE S1 BLAST sequence alignment output results using marker genes (ITS, *rbc*L, *tuf*A) of *B. sudetica* KNUA107.

Strain	Marker gene	Length (bp)	Closest match (GeneBank accession number)	Overlap (%)	Sequence similarity (%)	Taxonomic affinity
B.sudetica KNUA107	ITS	1092	*Botryococcus* sp. UTEX 2629 (AJ581914)	96	92	*Botryococcus* sp.
	*rbc*L	1423	*Botryosphaerella sudetica* UTEX 2629 (KC145508)	98	95	*Botryosphaerella sudetica*
	*tuf*A	929	*Neochloris aquatica* UTEX 138 (KT199248)	100	91	*Neochloris aquatica*

SUPPLEMENTARY TABLE S2 Fatty acid productivity and composition of *B. sudetica* KNUA107 under different mixotrophic cultivation conditions.

	BG11-12	BG11-30	Sucrose-12	Sucrose-30	Glucose-12	Glucose-30
C16:0	15.64 ± 0.68	24.54 ± 0.06	14.11 ± 0.08	18.98 ± 0.38	24.71 ± 0.40	27.66 ± 0.74
C16:1	5.44 ± 0.02	5.04 ± 0.18	6.59 ± 0.30	3.42 ± 1.00	6.84 ± 0.18	7.06 ± 0.47
C16:2	1.07 ± 0.22	1.76 ± 0.11	1.23 ± 0.04	1.52 ± 0.46	1.71 ± 0.17	1.04 ± 0.32
C16:3	20.57 ± 0.32	16.42 ± 0.04	23.90 ± 0.68	15.82 ± 0.17	9.92 ± 0.10	6.05 ± 0.16
C17:0	-	-	-	-	0.12 ± 0.06	-
C18:0	0.29 ± 0.00	0.83 ± 0.01	0.40 ± 0.03	0.56 ± 0.01	0.78 ± 0.01	2.12 ± 0.13
C18:1	7.06 ± 0.11	1.06 ± 0.02	8.12 ± 0.11	17.91 ± 0.34	26.55 ± 0.61	35.42 ± 0.51
C18:2	5.47 ± 0.23	7.06 ± 0.09	6.18 ± 0.08	5.55 ± 0.01	8.45 ± 0.01	5.12 ± 0.56
C18:3	38.38 ± 1.34	38.23 ± 0.90	36.01 ± 0.08	28.92 ± 0.68	18.72 ± 0.97	11.47 ± 0.33
C20:0	0.52 ± 0.37	-	-	-	-	-
C22:0	-	-	-	-	-	0.22 ± 0.11
SFA[a]	16.45 ± 1.05	25.37 ± 0.07	14.51 ± 0.11	19.54 ± 0.39	25.61 ± 0.47	30.00 ± 0.98
UFA[b]	77.99 ± 2.24	69.57 ± 1.34	82.03 ± 1.29	73.14 ± 2.66	72.19 ± 2.04	63.16 ± 2.35
TFA[c]	94.44 ± 3.29	98.23 ± 1.41	96.54 ± 1.4	92.68 ± 3.05	97.80 ± 2.51	93.16 ± 3.33

SFA[a] : Percentage of saturated fatty acids.
UFA[b] : Percentage of unsaturated fatty acids.
TFA[c] : Percentage of total fatty acids.
